# Mechanistic Understanding of the Effect of Obesity on Asthma and Allergy

**DOI:** 10.1155/2013/598904

**Published:** 2013-10-31

**Authors:** Anurag Agrawal, Akshay Sood, Allan Linneberg, Balaram Ghosh

**Affiliations:** ^1^Center of Excellence for Translational Research in Asthma & Lung Disease, CSIR Institute of Genomics & Integrative Biology, Delhi 110007, India; ^2^Division of Pulmonary and Critical Care Medicine, School of Medicine, University of New Mexico Health Sciences Center, University of New Mexico, Albuquerque, NM 87131, USA; ^3^Research Centre for Prevention and Health, Glostrup University Hospital, 57 Nordre Ringvej, 2600 Glostrup, Denmark

Obesity and asthma are two contemporaneous epidemics in the world today. Obesity is also a risk factor for asthma. This special issue highlights the mechanistic underpinnings of the increased risk of asthma in obese individuals. With increasing evidence that “obese-asthma” subjects respond poorly to conventional asthma treatment, this is an essential prelude towards the development of novel and innovative prevention and treatment strategies. The multiplicity of mechanisms by which obesity may cause or exacerbate asthma, or even vice versa, makes this a complex topic. This special issue compiles nine state-of-the-art papers, most of which are meticulously performed reviews of the available current literature that explore different aspects of this multidimensional subject. 

S. Farzan discusses whether there is a distinct asthma phenotype that is associated with obesity. Novosad et al. present the current understanding of the role of obesity in asthma control. It emerges that there are at least two distinct “obese-asthma” phenotypes, distinguishable mostly by age of onset. Early-onset obese asthmatics have increased atopy and eosinophilic airway inflammation, while the late-onset obese asthmatics show low eosinophilic airway inflammation despite being more symptomatic than their lean counterparts. Weight loss is associated with improved asthma control in some studies, and it seems that weight optimization may become an integral part of asthma control. 

Why obese subjects are at increased risk of asthma is addressed in five separate reviews, each illuminating a different facet. B. Brashier and S. Salvi focus on direct physiological consequences of truncal adiposity on lung function, such as reduced lung volumes and airway caliber and resulting increased airway hyperresponsiveness. F. Holguin summarizes the current understanding of dysfunctional arginine and nitric oxide (NO) metabolism in obesity and asthma. Elevated asymmetric dimethyl arginine (ADMA) in obese subjects competes with L-arginine and inhibits NO synthesis by uncoupling nitric oxide synthases, which may lead to oxidative stress and bronchoconstriction in lungs. This is an attractive hypothesis because the observed low exhaled NO in late-onset obese asthma is also associated with increased ADMA. U. Mabalirajan and B. Ghosh review the current understanding of the role of mitochondrial dysfunction in the pathogenesis of asthma. Since mitochondrial dysfunction is well known in obesity and the metabolic syndrome; this is an interesting mechanistic link that could be activated by a number of other pathways, such as increased ADMA. A. Sood and S. A. Shore discuss the population studies and basic research that reveal important roles for adipose tissue hormones like leptin, adiponectin, and resistin, in asthma pathogenesis. This provides valuable understanding of proinflammatory effects of adiposity in the context of asthma. S. Singh et al. explore available data on how insulin influences lung function and conclude that hyperinsulinemia may induce asthma-like changes in lungs. While insulin is not currently thought of as a major player in asthma pathogenesis, there are well-established effects of insulin on airway smooth muscle and fibroblasts in animal models that may promote bronchoconstriction and remodeling. Other papers present original research that advances our understanding of the roles of adiponectin and RhoA proteins in asthma, which may serve as novel targets for asthma treatment in the future. 

Despite large strides in our understanding of this complex topic, there are important limitations. It is unclear whether obesity-related metabolic changes such as increased ADMA, mitochondrial dysfunction, or hyperinsulinemia can also lead to increased asthma risk in absence of obesity. In many parts of the world, such as the Indian subcontinent, metabolic changes associated with obesity are commonly seen in nonobese individuals as well. If BMI-normal metabolically obese individuals are also at increased risk of asthma, an epidemic of even greater proportion lies ahead. While some insights have been gained from basic research and population studies, a clear picture is yet to emerge. We hope that this compilation of papers enriches the reader, spurs further research in these directions, and leads to novel management strategies for the “obese-asthma” phenotype.

*Anurag Agrawal* *Anurag Agrawal* *Akshay Sood* *Akshay Sood* *Allan Linneberg* *Allan Linneberg* *Balaram Ghosh* *Balaram Ghosh*

